# Crosslinked Collagenic Scaffold Behavior Evaluation by Physico-Chemical, Mechanical and Biological Assessments in an In Vitro Microenvironment

**DOI:** 10.3390/polym14122430

**Published:** 2022-06-15

**Authors:** Bianca-Maria Tihăuan, Gratiela Gradisteanu Pircalabioru, Mădălina Axinie (Bucos), Ioana Cristina Marinaș, Anca-Cecilia Nicoară, Luminița Măruțescu, Ovidiu Oprea, Elena Matei, Stelian Sergiu Maier

**Affiliations:** 1 Research & Development for Advanced Biotechnologies and Medical Devices, SC Sanimed International Impex SRL, 087040 Călugăreni, Romania; bianca.tihauan@sanimed.ro (B.-M.T.); madalina.axinie@sanimed.ro (M.A.); ioana.cristina.marinas@gmail.com (I.C.M.); smaier@tuiasi.ro (S.S.M.); 2 Research Institute of the University of Bucharest—ICUB, 91-95 Spl. Independentei, 50567 Bucharest, Romania; lumi.marutescu@gmail.com; 3 Academy of Romanian Scientists, Ilfov Street 3, 050054 Bucharest, Romania; ovidiu73@yahoo.com; 4 Department of Science and Engineering of Oxide Materials and Nanomaterials, Faculty of Applied Chemistry and Materials Science, University Politehnica of Bucharest, 1-7 Gh. Polizu Street, 011061 Bucharest, Romania; 5 Faculty of Pharmacy, University of Medicine and Pharmacy “Carol Davila”, 020021 Bucharest, Romania; anca.nicoara@umfcd.ro; 6 Faculty of Chemical Engineering and Biotechnologies, University of Politehnica Bucharest, Gh. Polizu Street 1-7, 011061 Bucharest, Romania; 7 National Institute of Materials Physics–Magurele, 405A Atomistilor Street, 077125 Magurele, Romania; elena.matei@infim.ro; 8 Department of Chemical Engineering in Textiles and Leather, Faculty of Industrial Design and Business Management, “Gheorghe Asachi” Technical University of Iasi, 700050 Iași, Romania

**Keywords:** collagen scaffold, inflammation, apoptosis, biocompatibility, wound healing

## Abstract

Wound healing-associated difficulties continue to drive biotechnological creativeness into complex grounds. The sophisticated architecture of skin wound sites and the intricate processes involved in the response to the use of regenerative devices play a critical role in successful skin regeneration approaches and their possible outcomes. Due to a plethora of complications involved in wound healing processes as well as the coordination of various cellular mechanisms, biomimetic approaches seems to be the most promising starting ground. This study evaluates the behavior of a crosslinked, porous collagen scaffold obtained by lyophilization and dehydrothermal reticulation (DHT). We address the key physio-chemical and mechanical factors, such as swelling, density and porosity, mechano-dynamic properties, SEM and TG-DSC, as well as important biological outcomes regarding scaffold biocompatibility and cellular metabolic activity, cytokine expression in inflammation, apoptosis and necrosis, as well as hemocompatibility and biodegradation. The mechanical and visco-elastic behavior are correlated, with the samples found to present similar thermal behavior and increased rigidity after DHT treatment. High biocompatibility rates were obtained, with no inflammatory stimulation and a reduction in necrotic cells. Higher percentages of cellular early apoptosis were observed. The hemocompatibility rate was under 2%, coagulation effects expressed after 4 min, and the DHT scaffold was more resistant to the biodegradation of collagenase compared with the untreated sample.

## 1. Introduction

Our skin is an intricate mantle with remarkable capabilities, with its appearance generally reflecting the health and effectiveness of its underlying structures. At a glance, it seems frail and slender, but its importance in our homeostasis is more than skin deep. Being the largest organ of our body, and a key component of the innate immune system, it plays the role of infantry in numerous cases, either against common invaders, such as UV radiation [1], superficial disruptions, allergens [2], heat [3] and various chemicals, or against far more elaborate attacks, such as burns [4,5], microbial infections and complications during wound healing processes [6,7]. Each type of attacks can make headway via its own route, leading to inflammation, irritation, skin tissue disruption, and, to top it off, this dysfunctional environment provides perfect conditions for microbial proliferation, which can lead to sepsis and permanent tissue damage. Such events mobilize our entire body to fight, but in many cases, without external support, success is unlikely to be achieved.

The microenvironment of a wound is highly refined by its own regulatory factors and its rich extracellular matrix (ECM), which determine the process of wound healing [8,9]. The complex macromolecules constituting the ECM include fibrous components (e.g., collagens and elastins) and glycoprotein components (e.g., fibronectin, proteoglycans and laminins). The intricate processes of tissue function, growth and repair are orchestrated by each one of these molecules’ interactions [10,11]. Wound repair is a complex process that involves sophisticated synchronization of a plethora of cell types with specific roles. These can be broadly categorized into the following four phases, which occur in a temporal sequence but are overlapping: hemostasis, inflammation, proliferation (cellular infiltration, angiogenesis and re-epithelialization) and maturation/remodeling [8]. The key steps of the wound healing process, such as hemostasis, inflammation and angiogenesis, are responsive to the ECM, collagen and its compounds [12,13,14,15]. 

Production of reproducible three-dimensional (3D) scaffolds represents one of the most important challenges in tissue engineering. Adequate biomimetic supports for cell migration and infiltration do not come easily, especially when they are required for inducing skin regeneration. In this case, the biomaterials should possess excellent biocompatibility, a suitable microstructure (e.g., mean pore size of 100–200 nm with >90% porosity), controllable biodegradability and suitable mechanical properties. Currently, in skin tissue regeneration biotechnologies, natural and synthetic polymers are sought after (e.g., cellulose derivatives, carrageenan and polyacrylates), as well as poly (vinyl alcohol, polyvinylpyrrolidone, silicones, alginate and collagen) [16]. Their importance lies in their capacity to improve the mechanical and biological functions of affected areas by providing suitable physical properties (e.g., a tensile modulus and pore structure) and, ideally, high-precision reproducibility. In spite of this, synthetic polymers are accompanied by several issues, such as toxicity or lack of recognition by cells, improper adhesion or the requirement for additional chemical modifications to enhance biological functions. On the other hand, natural polymers used as biomedical scaffolds have the advantage of being closely related to macromolecular substances, which the biological environment can recognize and accept into its metabolic processes [17]. Therefore, due to their outstanding biocompatibility and biodegradability, natural polymers such as collagen have been investigated as alternatives to synthetic scaffolds. Distinctly, collagen has been acknowledged for over a decade as the most promising scaffold material for applications in tissue engineering. Its popularity further confirms the level of importance, interest and the beneficial biomedical outcomes this protein has. A quick search on the National Center for Biotechnology Information (NCBI) database using the key words “collagen scaffold” with a filter for the last 5 years of publications resulted in 29,676 hits. It is safe to say that there are not many polymers that have received this level of dissection. 

One of the main advantages of collagen scaffolds is that they benefit wound healing by providing a skeleton of matrix for tissue types that are low in cellularity and have difficulty regenerating on their own after being affected by various external factors. The high porosity and generous cellular attachment sites of collagen in lyophilized scaffolds allow for the infiltration of additional cells, which aid remodeling. Porous scaffolds of lyophilized collagen may also present unique binding sites to cells that signal a remodeling response [18]. The mechanical properties (elasticity and reversible deformation) of fibrillar collagens are dependent on crosslinking processes. The nuances of crosslinking vary with the type of collagen and the tissue context and create a multi-layered hierarchical structure [19]. The composition and functionality of the collagen fibers influence the cellular response that is commonly regulated by integrin [20].

Other important advantages include the possibilities for customization of collagenic scaffolds in order to facilitate healing of numerous types of wounds, from superficial to chronic ones. By modifying each phase of fabrication based on the specific need of the tissues, bioscaffolds can be designed with improved therapeutic effects [21].

However, controlling collagen biodegradation and improving its mechanical properties remain challenging, and are current barriers to its implementation within large-scale production and clinical trials. Therefore, our study aims to assess the biomimetic behavior of a collagenic scaffold in an in vitro simulated tissue microenvironment. 

## 2. Materials and Methods

### 2.1. Collagenic Scaffold Preparation

#### 2.1.1. Chemicals and Enzymes 

All chemicals used were of analytical grade. The bovine Achilles tendon was provided by a local slaughterhouse, collected and stored in optional conditions for extraction. The other materials were sourced as follows: pepsin from porcine gastric mucosa (2000 FIP-E/g, 30000 E/g, 1:10000 NF, from Ascalia Pepsin, Seevetal, Germany); acetic acid glacial (≥99%, Sigma Aldrich, St. Louis, MO, USA); hydrochloric acid (37%, Sigma Aldrich)

#### 2.1.2. Collagen Extraction

Type I collagen was isolated from the bovine Achilles tendon (a by-product of the food industry). Collagen was isolated by the method of solubilization with non-specific enzyme (5.2% *w*/*w* pepsin solubilized in 0.02 M HCl solution, pH 1.8), in accordance with the methodology described in the literature [22]. This was followed by purification by diafiltration against purified water. Collagen can be easily obtained by chemical hydrolysis and enzymatic hydrolysis. Briefly, the tendon was cleaned with distilled water, cut into 1 cm pieces, frozen and then ground. Before the acidic hydrolysis was conducted, the tendon was thawed in the refrigerator overnight. Acid hydrolysis was performed by adding the thawed ground tendon in 0.2 M acetic acid solution, which was maintained for 24 h under constant stirring at 4 °C. At this stage, the chemical hydrolysis begins and the tendon doubles in volume, turning from opaque to transparent. For the extraction of collagen by enzymatic hydrolysis, the raw material from acid hydrolysis was added to 0.5 M acetic acid solution containing pepsin solubilized in 0.02 M chlorohydric acid. The mixture was continuously stirred for about 48 h at 15 °C followed by rough filtration and tangential filtration through a 0.8 μm filter, as for obtaining acid-soluble collagen. The purification and concentration of the extracted collagen was performed by diafiltration/ultrafiltration. The final collagen scaffold, hereafter referred to as Col-NT, was obtained by freeze-drying.

#### 2.1.3. Dehydrothermal Reticulation (DHT) 

The dehydrothermal reticulation process was performed as follows. The lyophilized collagen sponge was kept in a preheated vacuum oven (Heraeus Vacutherm VT 606) under vacuum (150 to 50 mbar), ranging the temperature during two days from 60 °C to 135 °C for a certain period of time and then cooled slowly to room temperature under vacuum, as shown in Table 1. The scaffold obtained following the heat treatment is hereafter referred to as Col-DHT.

According to Xuefei et al. [23], a collagen film obtained from steer hide showed favorable mechanical properties with DHT treatment at 125 °C for one day or 105 °C for five days. Increasing the DHT treatment time and temperature will damage to the triple helix structure and self-assembly of the collagen molecule. If the DHT treatment temperature is as high as 145 °C or the DHT treatment time exceeds five days, serious denaturation occurs.

### 2.2. Evaluation of Physico-Chemical and Mechanical Parameters

#### 2.2.1. Nitrogen Content and Total Protein Determination

The Kjeldahl method was used for total nitrogen content determination, using a conversion factor of total nitrogen to protein (6.25). The sample was digested in sulfuric acid (Merck, București, Romania) using CuSO_4_/TiO_2_ as catalysts, converting N to NH_3_, which was distilled and titrated. The percentage of protein in a sample was calculated according to the Equation (1):

(1) $$\mathrm{Protein}\;\mathrm{content}=\;\frac{\left(b-a\right)\cdot Ne\cdot 1.4007\cdot f}{Ws}\;\times \;n$$

were *b* is the volume of the 0.1 N sulfuric acid used in sample titration (mL); *a* is the volume of 0.1 N sulfuric acid used in blank titration; *Ne* is the expected normality of the sulfuric acid solution (0.1 N); *f* is the correction factor of the sulfuric acid normality obtained by using sodium carbonate solution; *Ws* is the weight (g) of the sample; *n* is the conversion sfactor of total nitrogen to protein (6.25). Samples of each kind were measured in triplicate.

#### 2.2.2. Moisture Content 

The moisture content was determined using Kern Moisture Analyzer DBS (Kern & Sohn, Balingen, Germany. The Moisture Analyzer determines the weight of the sample at the beginning of the measurement, after which the sample is heated with an inbuilt heating module and the moisture vaporizes. The equipment continually detects the sample weight and displays the reduction in moisture during the drying process. When the drying process is completed, the moisture content of the sample is displayed on the device screen. Samples of each kind were measured in triplicate.

#### 2.2.3. Swelling 

The swelling behavior of pure collagen was determined gravimetrically by immersing the sample in deionized water (DI) and an aqueous solution of urea (aq. urea) (6 M) at 30 °C for 5 days. In order to discourage bacterial proliferation, the immersion liquids were changed every day. After wiping the immersion liquid off the surface with a filter paper, the wet weight of the samples was measured. The samples were air-dried for 24 h to obtain the weight in a dry state. The swelling ratio was calculated using Equation (2):

(2) $$\mathrm{Swelling}\;\mathrm{Ratio}=\frac{W_{wet}-W_{dry}}{W_{dry}}\;\times \;100\%$$

where *W_wet_* and *W_dry_* are the wet and dry weights of the samples. Samples of each kind were measured in triplicate.

#### 2.2.4. Porosity and Density

The liquid displacement method was used to measure the porosity and density of the scaffolds. Ethanol was used as the displacement liquid. Collagen sponge scaffolds with a known weight (*W*) were immersed for 5 min in a cylinder containing 10 mL of ethanol (*V*_1_). The volume of the ethanol containing the sample was measured (*V*_2_) and the volume of the liquid was measured again after the removal of the scaffold (*V*_3_). The porosity of the scaffold was calculated using the Equation (3), while the density was calculated using the Equation (4):

(3) $$\mathrm{Porosity}=\frac{V_{1}-V_{3}}{V_{2}-V_{3}}\;\times \;100\%$$

(4) $$\mathrm{Density}=\frac{W}{V_{2}-V_{3}}\cdot \left[\frac{\mathrm{mg}}{{\mathrm{cm}}^{3}}\right]$$

Samples of each kind were measured in triplicate.

#### 2.2.5. Fluid Uptake Ability

The porous scaffolds were soaked in phosphate buffered saline (PBS) at room temperature for at least 5 min to reach equilibrious swelling. Subsequently, the sponge was removed for weighing after being gently blotted with a filter paper. The fluid uptake ability of the sample was calculated according to the following Equation (5):

(5) $$\mathrm{Fluid}\;\mathrm{uptake}\;\mathrm{ability}=\frac{m_{s}-m_{1}}{m_{1}}$$

where *m*_1_ is the weight of dried sponge and *m_s_* is the weight of swollen sponge. Samples of each kind were measured in triplicate.

#### 2.2.6. Water-Holding Capacity

The porous scaffolds were soaked in ultrapure water, and the swollen sponge was kept at 37 °C and 35% relative humidity in an incubator. At regular intervals of time, the scaffolds were removed and weighed. The weight remaining was calculated according to the following Equation (6):

(6) $$\mathrm{Weight}\;\mathrm{remaining}=\frac{m_{t}}{m_{0}}\;\;\times \;100\%$$

where *m*_0_ is the weight of swollen sponge and *m*_1_ is the weight of sponge at 30 min, and at 1, 2, 3, 4, 5, 6, 7 and 8 h. Samples of each kind were measured in triplicate.

#### 2.2.7. Fourier-Transform Infrared Spectroscopy (ATR-FTIR)

FTIR spectra of collagen scaffold samples were recorded with an Agilent Cary 630 FTIR spectrophotometer (Agilent, Santa Clara, CA, USA) in ATR mode, in the mid-infrared region, measuring the wavenumbers in the range between 4000–6500 cm^–1^ and were accumulated for 400 scans at 4 cm^−1^ resolution at 22 °C. 

#### 2.2.8. Morphological Characterization by Scanning Electron Microscopy

Analysis of surface morphological characteristics of samples Col-NT and Col-DHT was performed by scanning electron microscopy (SEM) analysis using a Zeiss Gemini 500 (Zeiss, Oberkochen, Germany). 

#### 2.2.9. Mechanical and Dynamic Mechanical Analyses (DMA)

Stress–strain dependence was recorded using the DMA 242 Artemis apparatus from Netzsch (NETZSCH-Gerätebau GmbH, Selb, Germany), equipped with a sample holder for compression (pushrods made of fused silica), in compression mode, at room temperature, under a linear increase of compression force of 50 μN/min, at a total load varying between zero and 8 mN. DMA investigation was performed on the same apparatus, equipped with a sample holder for sharing (in sandwich geometry; clamps and pushrod with grooved surface), on samples of 15 mm diameter and 5 mm height. Flexural moduli (E′, E″) and the dissipation factor (tan δ) were measured in temperature scan mode, under heating between −100 and +280 °C with a linear increase of 2 °C/min. The mechanical oscillations were induced at a frequency of 1 Hz, under a dynamic stress of 65 kPa (in the Hooke’s linear viscoelastic range of the samples, identified on the stress–strain curves).

#### 2.2.10. Thermogravimetry–Differential Scanning Calorimetry (TG-DSC)

The thermal analysis TG-DSC for the Col-NT and Col-DHT samples was performed with a Netzsch STA 449C Jupiter (NETZSCH-Gerätebau GmbH, Selb, Germany). The samples were placed in an open crucible made of alumina and heated with 10 K·min^−1^ from room temperature up to 900 °C, under a flow of 50 mL min^−1^ dried air. An empty alumina crucible was used as reference.

### 2.3. Assessment of Biological Response to Implantable Collagenic Scaffold 

#### 2.3.1. In Vitro Cytotoxicity Analysis 

HEp-2 human epithelial cells (adenocarcinoma) (ATCC-American Type Culture Collection) were selected as the model for cytotoxicity assessment. MTT assay was used for measurement of cellular metabolic activity and as an indicator of cell viability, proliferation and cytotoxicity. HEp-2 cells were cultivated in DMEM culture medium (Sigma-Aldrich, St. Louis, MO, USA) supplemented with 2 mM Glutamine (Sigma-Aldrich), 10% Fetal Bovine Serum (FBS) (Sigma-Aldrich) and 1% Pen/Strep (penicillin /streptomycin solution, 50 µg/mL, Sigma-Aldrich) for 24 h at 37 °C and 95% humidity with 5% CO_2_. After 24 h, cells were washed with PBS (Phosphate Buffered Solution, Sigma-Aldrich), harvested using trypsin (Sigma-Aldrich) and counted using Trypan Blue (Sigma-Aldrich) and a hemocytometer. The seeding density for the MTT assays was optimized at 4 × 10^5^. Cells seeded at 4 × 10^5^ density in a clear 24-well cell culture plate were treated with collagenic samples and controls and incubated for 24 and 48 h at 37 °C and 95% humidity with 5% CO_2_. After 24 and 48 h of exposure to tested compounds, cells were incubated for 4 h with MTT reagent (Roche, Basel, Switzerland) at 37 °C and 95% humidity with 5% CO_2_. After incubation, cells were treated with MTT solvent (Roche) for 15 min at room temperature. Absorbance was measured using a spectrophotometric microplate reader (ELISA reader, Thermo Scientific, Waltham, MA, USA) at OD = 570 nm. With the LDH Cytotoxicity Detection Kit (Roche), LDH activity was measured in culture supernatants using a spectrophotometric microplate reader (ELISA reader) at 492 nm with a 600 nm wavelength reference.

#### 2.3.2. Assessment of Inflammatory Cytokine Panel 

RAW 264.7 murine macrophages were used in order to evaluate the immune response induced by the collagen scaffolds. Cells were grown in DMEM medium supplemented with 10% fetal bovine serum. Positive controls were represented by macrophages stimulated with two bacterial strains (10^6^ colony forming units/mL)—*E. coli* (ATTC strain) and *S. aureus* (ATCC). After 24 h of stimulation, either with the collagenic material or with the bacterial strains, the supernatants were collected for cytokine quantification. Cytokine analysis was performed as per the manufacturer’s instructions (Mouse Luminex screening Assay, R&D, Shanghai, China). The fluorokine beads (IL-1β, IL-6, IFN-γ and IL-17) were diluted 1:100 in bead dilution buffer. Next, 50 μL of diluted bead was added to 50 μL of the sample in a 96-well filter bottom plate. Loaded 96-well plates were incubated overnight at 4 °C on an oscillating rocker (60 Osc./min). After washing, biotinylated primary antibodies (1:100 dilution) and plates were incubated at room temperature, protected from light (2 h at 60 Osc./min). After washing off the primary antibody, streptavidin-PE (1:100 dilution) was added to each well and plates were incubated (1 h, 70 Osc./min, protected from light). Finally, 100 μL of wash buffer was added to each well and samples were analyzed using a Luminex xMAP200 (Luminex, Austin, TX, USA). Data points are represented as mean fluorescence intensities (MFI).

#### 2.3.3. Evaluation of Early/Late Apoptosis and Necrosis 

Determination of early and late apoptosis was performed by flow cytometry using a FACS Alexa cytometer and an Alexa Fluor^®^ 488 Annexin V/Dead Cell Apoptosis Kit (Thermo Fisher Scientific, Waltham, MA, USA). In total, 10,000 cells were analyzed per measurement. Data were analyzed using FlowJo 10.0.7 software (Tree Star Inc., Ashland, Wilmington, DE, USA). HEK-293 T cells were cultured in DMEM medium supplemented with 10% FBS under standard cell culture conditions (37 °C, 5% CO_2_). Cell numbers were assessed using a hemocytometer. Trypsin/EDTA (0.05%, Invitrogen, Waltham, MA, USA) was employed to detach the cells from the flask, either for passaging, heat shock induction or flow cytometry. For induction of apoptosis, cells were exposed to room temperature (22 °C) for a period of 24 h to collagenic scaffolds. HEK-293 T cells (3 × 10^6^) were stained using the Alexa Fluor^®^ 488 Annexin V/Dead Cell Apoptosis Kit according to manufacturer instructions. Stained cells were diluted in Annexin V-binding buffer. Suspended cells were used to perform the flow cytometry assay. 

#### 2.3.4. Hemolysis Ratio

The hemolysis ratio was analyzed using citrated whole blood diluted with physiological saline solution according to Balaji et al. [24]. The samples were equilibrated in physiological saline (0.9% *w*/*v*) at 37 °C for 30 min. Then, they were incubated in the presence of citrate blood and diluted with physiological saline solution (4:5) for 1 h at 37 °C. Subsequently, the whole blood was diluted with distilled water to cause complete hemolysis (positive control) and also with physiological saline (negative control). After incubation, the samples were centrifuged at 5000 rpm for 15 min. The absorbance was measured at 524 nm for the supernatant to record the amount of hemoglobin released. The percentage of hemolysis (HR%) was calculated using the formula:

$$\mathrm{HR}\;\left(\%\right)=\frac{A\;\mathrm{sample}-A\;\mathrm{negative}\;\mathrm{control}}{A\;\mathrm{positive}\;\mathrm{control}-A\;\mathrm{negative}\;\mathrm{control}}\;\times \;100$$

#### 2.3.5. Hemoglobin Absorption

The hemoglobin adsorption assay was conducted according to Yan et al. [25]. A quantity of 10 μL whole blood solution (per 10 μL blood with 0.8 μL of 0.2 M CaCl_2_) was added onto the sponge surface in a glass dish and incubated for 1, 2, 3, 4 and 5 min at 37 °C. The negative control involved the addition of whole blood to the glass dish. Then, 2 mL of distilled water was added to dissolve the hemoglobin in free hemocytes. The content of hemoglobin on the samples was measured using a UV-Vis spectrophotometer at 542 nm by the following equation:

$$\mathrm{Hemoglobin}\;\mathrm{adsorption}\;\mathrm{rate}\;(\mathrm{HAR}\%)=\frac{\mathrm{Ir}\;–\;\mathrm{Is}\;}{\mathrm{Ir}}\;\times \;100\%$$

where, Is is the absorbance of hemoglobin in suspension and Ir is the absorbance of the reference value. As a reference value, 10 μL of unreacted blood was dropped directly in 2 mL of distilled water to measure its absorption value.

#### 2.3.6. Collagenase Assay

The method was performed according to the protocol described by Sun et al. [26] with minor modifications. Briefly, 5 mg of the sample was weighed, then immersed in 0.1 M Tris-HCl (pH 7.4) containing 50 mM CaCl_2_ at 37 °C for 1 h. Then, the biodegradation was performed at 37 °C for 24 h after the addition of 200 U bacterial collagenase *Clostridium hystolyticum* (Serva Electrophoresis GmbH, Heidelberg, Germany). Digestion was completed by adding 0.25 M EDTA and cooling on ice. The samples were centrifuged for 15 min at 4 °C, and the hydroxyproline was determined for supernatants by the method described by Kolar [27]. A standard curve was plotted by using different concentrations (1–200 µg/mL) of hydroxyproline (R2 = 0.9991). 

### 2.4. Statistical Analysis 

The statistical analysis was performed using GraphPad Prism 9 (San Diego, CA, USA). For biocompatibility, the data were analyzed using the two-way ANOVA test. All data were expressed as the mean ± SD for triplicates. For hemocompatibility, data were analyzed using ordinary two-way ANOVA with the two-stage linear step-up procedure of Benjamini, Krieger and Yekutieli, with individual variances computed for comparison of Col-NT and Col-DHT. For biodegradability (%) the analysis was performed using the unpaired t-test method. A value of *p* < 0.05 was considered to be statistically significant.

## 3. Results

### 3.1. Evaluation of Physico-Chemical Parameters

#### 3.1.1. Nitrogen Content and Total Protein Determination 

Protein concentration was determined for samples of the untreated collagen scaffold (Col-NT) and the dehydrothermally treated collagen scaffold (Col-DHT) using the Kjeldahl method. A nitrogen content of 14.77% was recorded for the untreated collagen scaffolds, leading to a total protein content of 92.35% (Table 2). The dehydrothermal reticulation process did not affect the total protein content, which was 93.44%. 

#### 3.1.2. Moisture Content

The moisture content was much lower in the case of Col-DHT (5.17%) compared with the Col-NT (13.23%) (Table 2). The water in the collagen scaffold, both free and hydrogen-bonded water, was substantially eliminated during the dehydrothermal treatment of heating under a vacuum. 

#### 3.1.3. Swelling

The DHT treatment causes crosslinking, which improves the mechanical properties of scaffolds. As a result, it was important to understand the type of crosslinking caused in the treated samples.

Protein denaturants such as urea are commonly used to analyze protein stability. Julian at al. [28] found, by molecular dynamics simulation, that urea disrupts the original hydrogen bonding system. At the same time, urea forms hydrogen bonds with polar residues of the peptide backbone.

In a high-concentration aq. urea solution, the protein’s noncovalent hydrogen bonding is broken, and the protein swells significantly [29]. The covalent crosslinking in the protein, on the other hand, protects it against urea assault and more strictly maintains the protein’s integrity. Both types of crosslinking can exist within a swollen collagen scaffold in DI water. By comparing the swelling ratio of the collagen scaffold in DI water and in aq. urea, the fractions of covalent and noncovalent crosslinks can be determined.

The swelling ratios of the collagen scaffold in DI water and urea, before and after applying the DHT treatment, are shown in the Table 2. The lowest swelling ratio in water for the untreated collagen scaffold was 5.5, while in urea it was 15.5. The results indicate that both hydrogen and covalent bonded crosslinks exist in the collagen scaffold, since urea breaks the hydrogen bonding in the structure. 

The swelling ratio in DI water for thermally treated collagen was reduced to 2.9, indicating that extra crosslinking was introduced. In contrast, the swelling ratio in urea was reduced considerably to 3.5, almost reaching the same value as the swelling ratio in water, indicating that the crosslinking upon dehydrothermal treatment mostly comprises covalent bonding, without excluding the formation of hydrogen bonding.

#### 3.1.4. Porosity and Density

Products that are to be used as medical devices and applied directly to wounds have to absorb all hematic and lymphatic fluids present in the wound itself. From this point of view, the porosity and the density of the collagen scaffold play important roles. The results of the porosity and density analyses are presented in the Table 2. The porosity of the Col-DHT was lower than that of the Col-NT.

#### 3.1.5. Fluid Uptake Ability

The scaffolds were easily wettable by polar solvents such as PBS. They exhibited a high swelling ability; this is because collagen contain a large number of functional groups capable of binding water. This fast-swelling behavior is a characteristic property of hydrophilic and porous materials. PBS solution has a pH of 7.4, which corresponds to the pH of blood. The use of such a solution allows for examination of the behavior of the material after its application inside the body. The percentage of scaffold swelling after immersion in PBS for 1 h, together with the results of the moisture content measurements, are presented in Table 2.

#### 3.1.6. Water Holding Capacity

**Figure 1:**
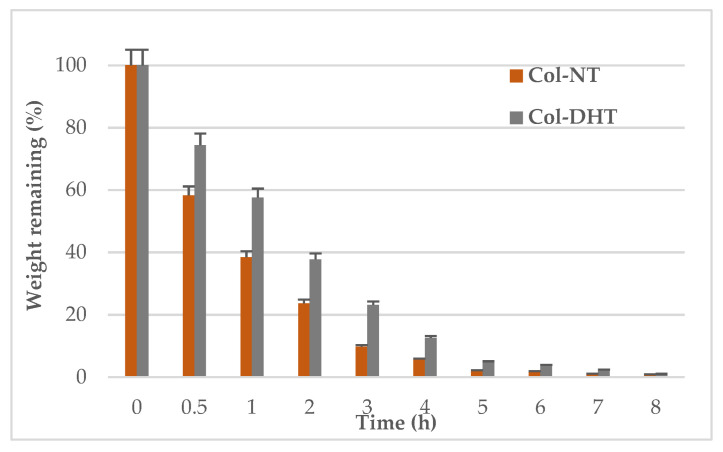
Water holding capacity of 
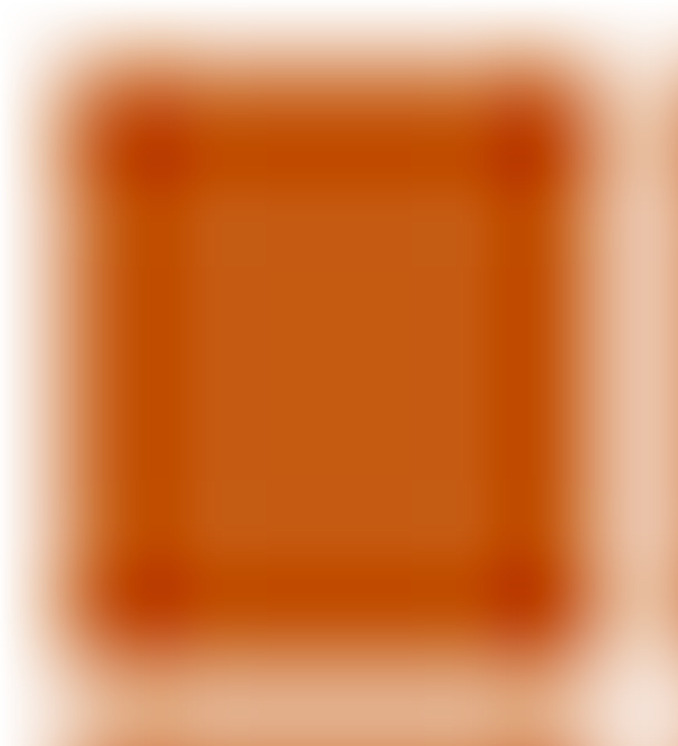
 untreated and 
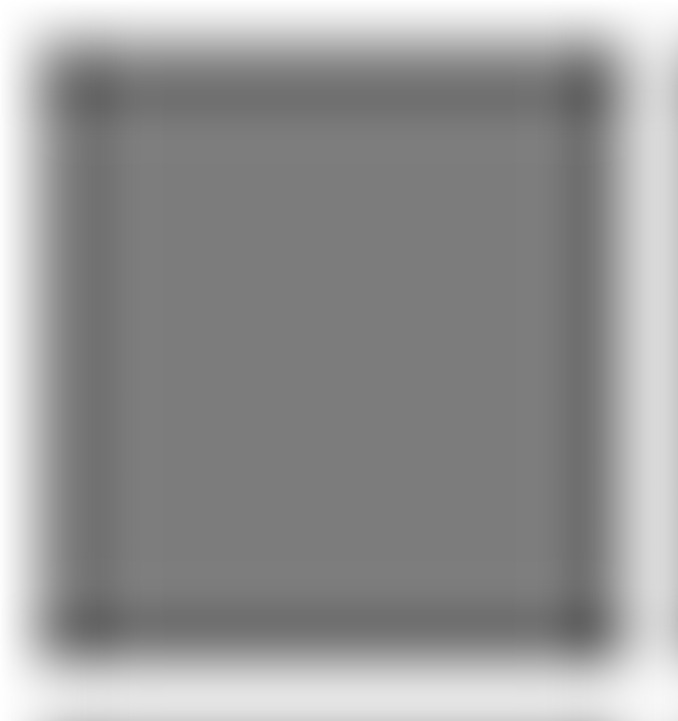
 dehydrothermally treated collagen scaffolds.

The water holding capacity was evaluated for both scaffolds. Based on the weight remaining percentage over time, it was found that the Col-DHT scaffold lost water more slowly than the Col-NT (Figure 1). After 5 h, the Col-NT scaffold had lost all of the water, whereas this process took 7 h in the case of the Col-DHT scaffold.

#### 3.1.7. Fourier-Transform Infrared Spectroscopy (ATR-FTIR) 

**Figure 2:**
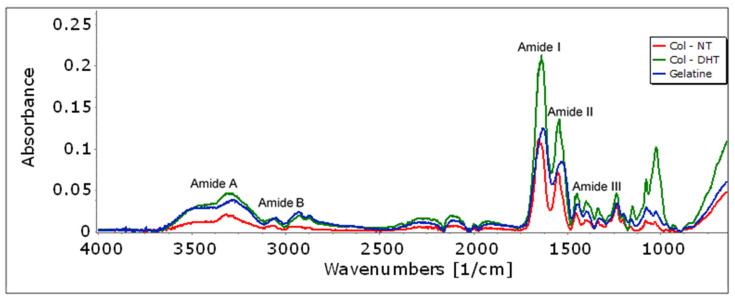
FT-IR spectrum of native collagen (Col-NT), dehydrothermally treated collagen (Col-DHT) and gelatin.

The changes in the chemical structure of the collagen that occurred upon DHT treatment were analyzed using Fourier-transform infrared spectroscopy. Significant information can be extracted from Figure 2, which illustrates the characteristic bands distribution of native collagen (Col-NT): Col-NT exhibits relatively low average absorbance in the region 800–1200 cm^−1^, moderate absorbance at 1300–1500 cm^−1^ and high absorbance in the regions 1500–1700 cm^−1^ and 2800–3500 cm^−1^. The amide I band associated with the stretching vibration of the carbonyl group along the polypeptide backbone was found at 1653 cm^−1^. The amide II and III bands were observed at 1550 cm^−1^ and 1238 cm^−1^, and are associated with N-H bending vibrations and C-H stretching, respectively. The amide A band was found at 3317 cm^−1^ and is associated with N-H stretching vibration and hydrogen bands [30]. The amide B band was found at 2952 cm^−1^ and is related to the asymmetrical stretching of CH_2_.

In order to determine the changes in the amide I band during the dehydrothermal treatment of collagen (Col-DHT), the IR spectra for both pure collagen (Col-NT) and gelatin were recorded. Gelatin is a hydrolyzed form of collagen with a similar chemical composition, but which partially or completely loses its native triple helix structure. The variation of the amide I band was clear during the thermo-denaturation process. The center of the amine I band of the Col-DHT (1637 cm^−1^) spectra shifts to lower wavenumber compared with the Col-NT (1653 cm^−1^) but does not reach the values corresponding to gelatin (1630 cm^−1^); this proves that, during the thermal treatment, a series of changes occurs in the collagen triple helix structure, but these do not lead to a complete denaturation.

In addition, the effects of crosslinking on the amide and ester functional groups were analyzed using FTIR spectra. As a result of the thermal treatment, the peak height at 1550 cm^−1^ increases, indicating an increase in the number of amide bonds [31], corresponding to an increased crosslink density; this means that the free –NH_2_ groups in collagen were converted to N-H groups. Analysis of the IR spectrum around 1152 cm^−1^ shows an increase in the number of ester bounds due to the thermal treatment.

#### 3.1.8. SEM Analysis 

**Figure 3:**
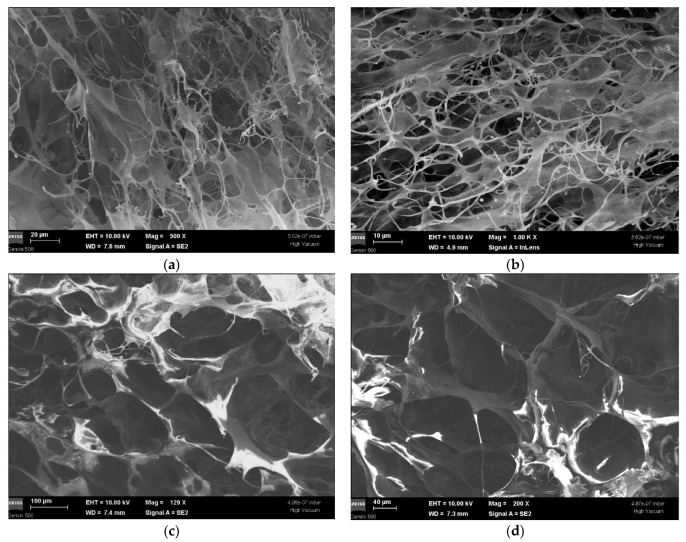
Monographs of sample Col-NT (**a**,**b**) and Col-DHT (**c**,**d**).

Figure 3a,b depicts SEM micrographs of the control collagen matrix (Col-NT) at different scales (20 and 10 µm), in several areas of the scaffold, in order to highlight (at magnitudes between 500 × and 1.00 k ×) its specific structural aspects, namely the porosity and the frail appearance of the fibers, arranged in a network-type structure. The pore size varies from 40 nm to 200 nm. 

Monographs 3c,d at 40 and 100 µm scales and magnitudes of 129 × and 200 × represent the aspect of sample Col-DHT. The porosity and collagen fiber arrangement can be seen, and the rigid network structure (in comparison with Col-NT) can be observed. The pore size (varying from 54 nm to 265 nm) is visibly modified as compared to Col-NT, appearing condensed and well-contoured, and the rigidity of the scaffold seems to be increased. 

#### 3.1.9. Mechanical and Mechano-Dynamic Properties

**Figure 4:**
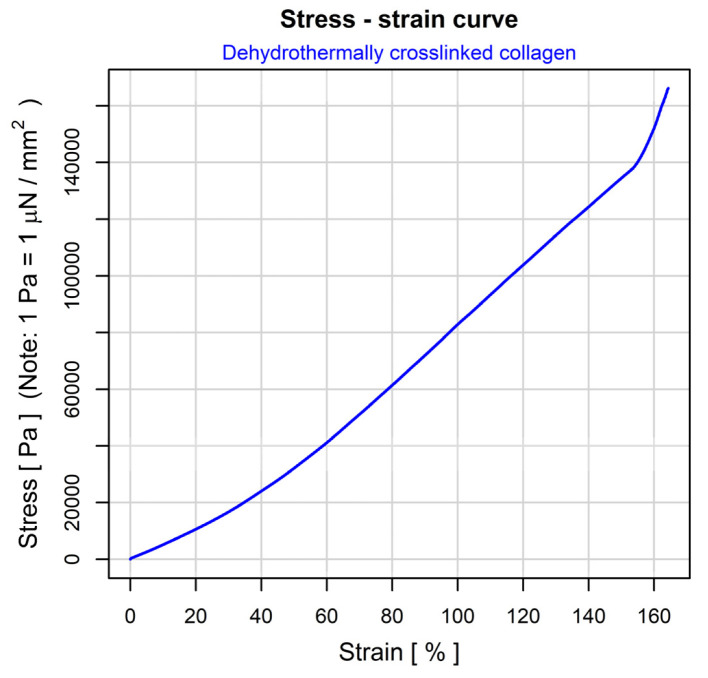
The stress–strain curve for dehydrothermally crosslinked collagenous scaffold.

**Figure 5:**
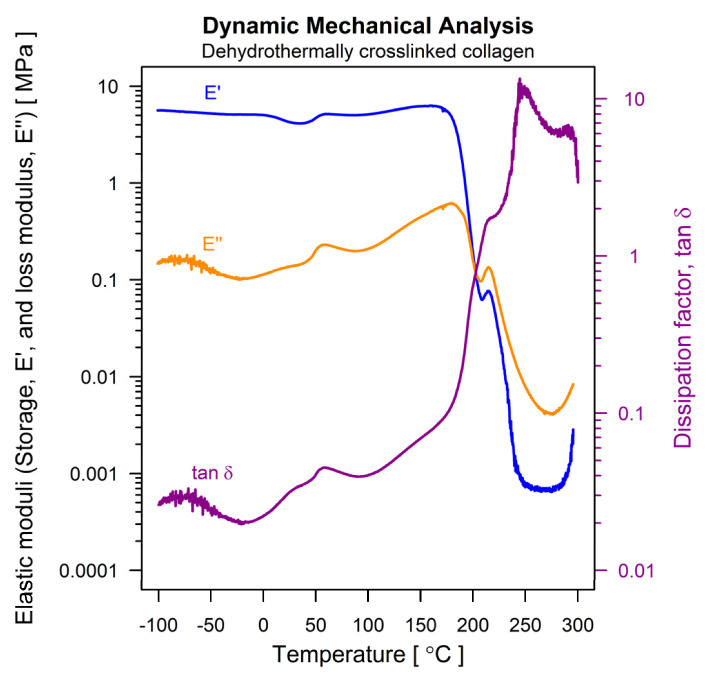
Thermo-mechanical behavior of the dehydrothermally crosslinked collagenous scaffold, expressed by the elastic moduli and the dissipation factor.

The physical-mechanical behavior of the dehydrothermically crosslinked collagen scaffolds was studied according to a protocol designed for insulating foams, in two steps: (i) identification of the linear visco-elastic domain by means of a stress–strain experiment, and (ii) determination of visco-elastic properties by subjecting the sample to an oscillating force of a precise value. For both types of experiments, the applied forces and the resulted deformations were accurately measured, and the physical-mechanical properties of the scaffold material were then calculated. Figure 4 depicts the result of the stress–strain experiment. Three domains can be identified. The first two (0 to 60% strain and 60 to 150% strain) follow conventional patterns (nonlinear rearrangements and linear sliding of fibrous elements, respectively), but the pattern of third domain (beyond 152% strain) is unusual, probably due to the progressive rupturing of the elongated fibers. The smallest feasible strain to fall within the linear domain of the stress–strain curve is 65%. This value was used to conduct the second step of the mechano-dynamic investigation. Figure 5 describes the thermally induced evolution of the main visco-elastic parameters of the scaffold material: the storage modulus (E′) and the loss modulus (E″), together with their ratio (E″/E′) and the dissipation factor, tan δ. The expected evolution was registered in the domain between −50 … +100 °C, where “melting” of the triple-helical structure of collagen occurs, at around 60 °C. At higher temperatures, a progressive destruction of the collagen fibrous network occurs, and the scaffold material apparently begins to flow, until physical destruction begins, at around 280 °C. A short-range network rearrangement seems to be promoted between 210 and 220 °C, probably due to the complete breakdown of the dehydrothermally induced crosslinkings. 

#### 3.1.10. Thermogravimetry–Differential Scanning Calorimetry (TG-DSC)

The samples exhibited a similar thermal behavior. In the RT-160 °C range, the samples lost ~10% of their initial mass, most likely due to the elimination of residual water molecules (evaporation of water molecules bound by strong hydrogen bonds, responsible for the stability of the triple helix) (Table 3). 

**Figure 6:**
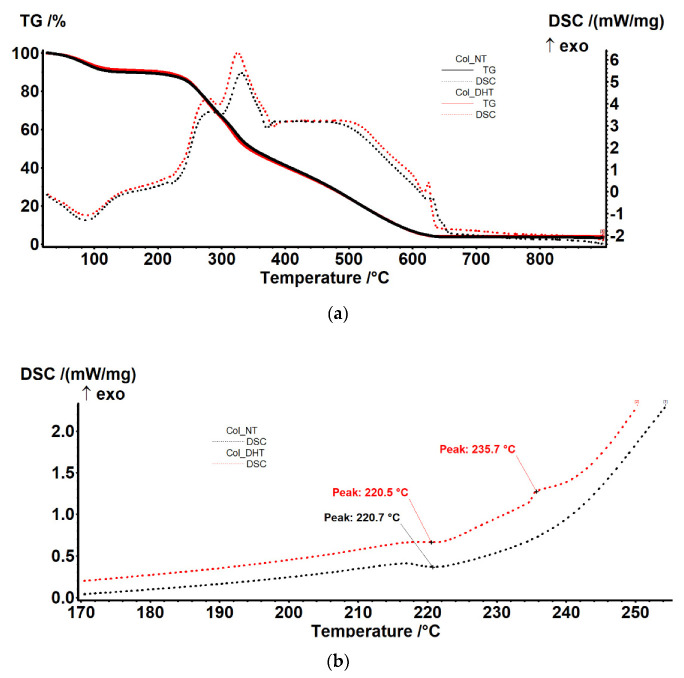
Thermal behavior of samples of Col-NT and Col-DHT (**a**); detail of DSC curves (**b**).

This process was accompanied by an endothermic effect with a minimum of 87.3 °C. The samples underwent oxidative degradation after 200 °C, losing ~87% of their mass up to 700 °C (Figure 6a). 

The degradation process occurred in three phases, partially overlapping, as can be seen from the exothermic effects on the DSC curve. In the first part, the oxidation of the lateral groups, labile at ~280 °C, probably occurred, followed by the oxidation of the main chain at ~325 °C. At higher temperatures, the oxidation process would have been continuous, slow and accompanied by a wide exothermic effect between 380–490 °C. Around 625 °C there was a sharp exothermic effect that can be attributed to the presence of sodium in the sample or the burning of carbon dioxide. Detailed analysis of the DSC curve (Figure 6b) indicates the presence of a weak endothermic effect at 220 °C, corresponding to the transition from triple-helix to an accidentally twisted chain. This transition is specific to collagen, and occurs when hydrogen bonds are broken by rising temperatures, turning the collagen into gelatin.

### 3.2. Assessment of Biological Response to Implantable Collagenic Scaffold

#### 3.2.1. In Vitro Cytotoxicity Analysis 

**Figure 7:**
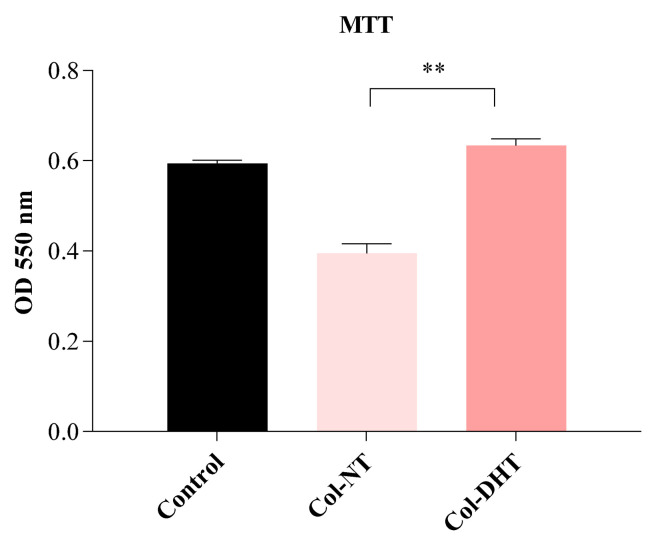
Evaluation of cellular biocompatibility of Col-NT (collagenic scaffold no-treatment) and Col-DHT (collagenic scaffold dehydrothermally reticulated) samples using MTT assay on HEp-2 cells. The assay was performed in triplicate (*n* = 3); ** = 0.001.

The HEp-2 cell line was used to evaluate the biocompatibility of the collagenic scaffolds. An MTT assay was performed to evaluate cellular metabolic activity and an LDH assay to evaluate cytotoxicity and consequently extensive membrane damage. The results obtained by MTT assay (Figure 7) indicate that the Col-DHT samples had high viability rates (>95%) in comparison with the control samples represented by untreated cells, and fairly lower rates that for the Col-NT (<65%), indicating that the dehydrothermal reticulation provided a better microenvironment for cellular proliferation. Obtaining an extensive network of stable intermolecular crosslinks for the sample Col-DHT considerably improved its biocompatibility compared with the original, non-treated collagenic scaffold. 

**Figure 8:**
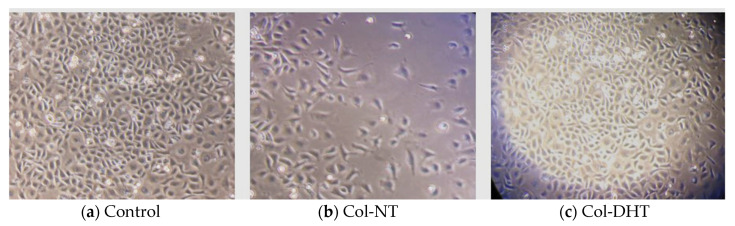
Depiction of cellular adherence and viability after contact with samples Col-NT and Col-DHT—phase contract microscopy, 20X; (**a**) HEp-2 cells control after 24 h culture time; (**b**) sample Col-NT after 24h of contact with HEp-2 cells; (**c**) sample Col-DHT after 24 h of contact with HEp-2 cells.

Assessment of cellular behavior after 24 h of contact with each collagenic scaffold using a phase contrast microscope (Figure 8) enabled us to correlate the results obtained during the MTT and LDH tests, indicating good adherence for the control sample and the Col-DHT sample, with confluence over 80%, normal proliferation rates specific to this cell line, and, consequently, lower adhesion for the Col-NT sample. In this case, cellular growth appeared to be inhibited. A significant difference in cell growth can be observed between the Col-NT and Col-DHT.

**Figure 9:**
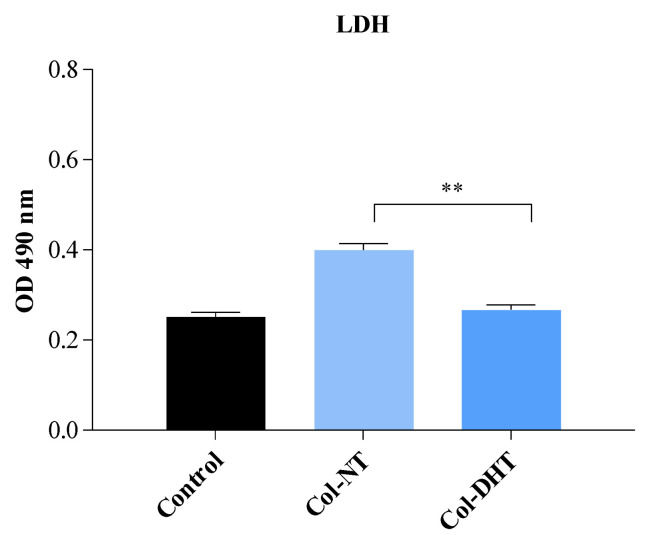
Evaluation of cellular cytotoxicity of Col-NT (collagenic scaffold no-treatment) and Col-DHT (collagenic scaffold dehydrothermally reticulated) samples using LDH assay on HEp-2 cells. The assay was performed in triplicate (*n* = 3); ** = 0.001.

The evaluation of LDH activity can indicate intra- and extra-cellular values of pyruvate, and quantitatively measure cytotoxicity in response to applied treatments. The results obtained (Figure 9) indicate increased values of LDH release for sample Col-NT (15% higher than control) and values similar to the control for the sample Col-DHT. These findings, in correlation with the results obtained in the MTT assay and phase contrast images, indicate membrane damage as a result of exposing cells to the Col-NT sample.

#### 3.2.2. Assessment of Inflammatory Cytokine Panel

In the context of skin wounds, proinflammatory cytokines are among the first factors to be produced in response to barrier damage, and they regulate the functions of immune cells in epithelialization. Proinflammatory cytokines mainly include interleukins (IL)-1β, IL-6 and interferon (IFN-γ), among others, and participate in the inflammation phase of wound healing through activating downstream cascades [32]. Cytokines play a tremendous and crucial role in the epithelialization phase by mobilizing resident stem cells and promoting cell proliferation and differentiation [33]. It should be kept in mind that the extent of immune responses in wound healing can be quite complex: although moderate immune responses promote wound healing, and normal levels of proinflammatory cytokines therefore prevent infection and accelerate normal wound healing, excessive production of proinflammatory cytokines is detrimental, and it possibly results in deregulated activation and differentiation of epidermal stem cells [34]. 

**Figure 10:**
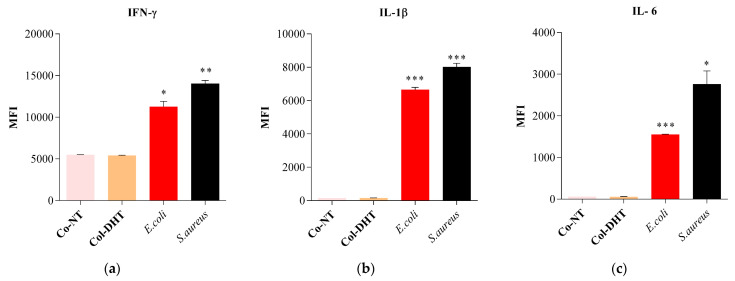
Differential expression of murine cytokines (**a**) IFN-γ; (**b**) IL-1β; and (**c**) IL-6 after treatment for 24 h with samples Col-NT and Col-DHT. The assay was performed in triplicate (*n* = 3); * = 0.012; ** = 0.002; *** = 0.0005.

RAW murine macrophages were exposed to samples Col-NT, Col-DTH, *E. coli* and *S. aureus* (as positive controls). The results obtained for IFN-γ (Figure 10a) indicate similar values for both collagenic scaffold samples, with decreased expression levels in comparison to the *E. coli* and *S. aureus* controls. The IL-1β and IL-6 values (Figure 10b,c) obtained for the samples were significantly decreased in comparison with those of the *E. coli* and *S. aureus* samples. The cytokine levels observed after 24 h treatment with each sample indicate a non-stimulatory effect on the immune response, with no clear distinction between the two types of collagenic scaffolds, suggesting that the dehydrothermal reticulation did not impact cytokine expression in any way. 

Regarding the immune response to the wound healing scaffolds, it is important to mention that inadequate proinflammatory signaling can result in wounds that take much longer to heal and are at great risk of infection [34,35,36]. In this context, lower rates of IL-1β expression are associated with stepping from proinflammatory conditions to healing and increased levels of growth factors [37]; as for IL-6, its signaling role in the inflammation process is responsible for the switch to a reparative environment [38]. Being mainly secreted by CD4+ T helper 1 (Th1), natural killer (NK) and NKT cells after skin injury, IFN-γ, is recognized as an inhibitory factor for collagen synthesis [39], and its role in inhibiting the proliferative phase has been previously identified [40]. It also plays a role in the suppression of the inflammatory response (with great importance for patients with autoimmune diseases) [41,42]. 

#### 3.2.3. Evaluation of Early and Late Apoptosis and Necrosis 

The quantitation of apoptosis and necrosis was performed by assessing annexin V binding and propidium iodide uptake using flow cytometry. This procedure allows analysis of multiple parameters of cell health and provides a high throughput analysis of cytotoxicity and cellular behavior. Apoptosis and necrosis occur during cell death in response to cytotoxic conditions. Cell death can occur either by apoptosis, a highly regulated biochemical pathway involving signal transduction cascades, or by necrosis. Necrosis is accompanied by mitochondrial swelling and increased plasma membrane permeability, while apoptosis involves an articulated breakdown of the cell into membrane-bound apoptotic bodies [43]. There are a number of assays that are designed to measure cytotoxicity and cell death, independent of their mechanisms. Most of these assays assess cell viability by measuring plasma membrane permeability using specific fluorochromes.

It is important to mention that the measurement of cell death by Annexin V binding and PI uptake depends intimately on the phosphatidylserine (PS) response. PS is located on the inner side of the intact plasma membrane of live cells and these cells do not stain with Annexin V or PI. PS localization is turned over to the outer side of the plasma membrane of apoptotic cells and these cells bind Annexin V on the outside but still exclude PI. Annexin V binds to PS on the plasma membrane and PI is taken up and binds to the DNA [44]. 

**Figure 11:**
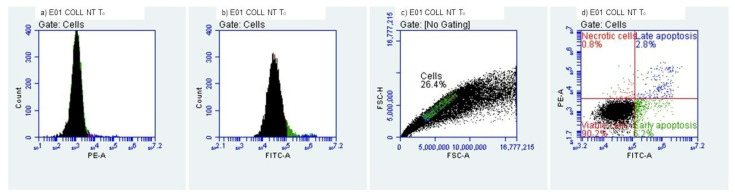
Collagenic scaffold non-treated, T_0_ Initial contact with HEK-293 T cells.

**Figure 12:**
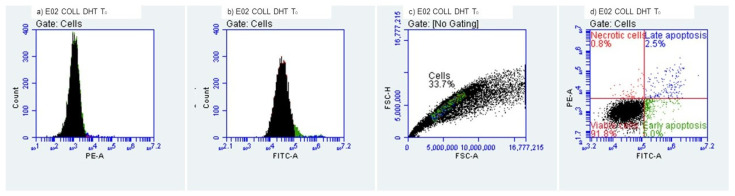
Collagenic scaffold dehydrothermally reticulated, T_0_ Initial contact with HEK-293 T cells.

We evaluated the early and late apoptosis and necrosis expression after treating HEK-293 cells with the collagenic scaffolds. At T_0_, i.e., the initial contact point (Figure 11 and Figure 12), viability percentages were over 90%, results that correlate with the MTT and LDH findings. The early apoptosis was 6.19% for the Col-NT sample and 4.95% for the Col-DHT sample. Late apoptosis presented at 2.80% for Col-NT and at 2.45% for Col-DHT (Table 4); as for the necrosis expression, lower rates were observed for both samples (0.82% for Col-NT and 0.76% for Col-DHT). The results obtained at this stage indicate that Col-DHT presented more favorable outcomes in comparison with the Col-NT sample, as it induced lower rates of apoptotic and necrotic factors. 

**Figure 13:**
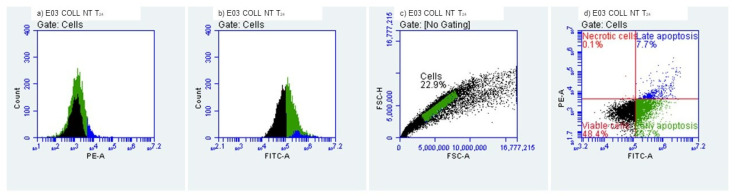
Collagenic scaffold non-treated, T_24h_ Time lapsed after contact with HEK-293 T cells.

**Figure 14:**
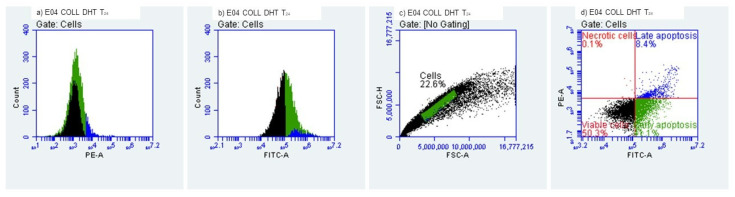
Collagenic scaffold dehydrothermally reticulated, T_24h_ Time lapsed after contact with HEK-293 T cells.

At T_24h_ after contact with HEK-293 T cells (Figure 13 and Figure 14), decreased levels of viability were observed, with 48.44% for sample Col-NT and 50.35% for sample Col-DHT, indicating a more than 50% loss in cellular metabolic activity. Early apoptosis was present in 43.70% of the cells exposed to Col-NT and 41.11% exposed to Col-DHT. Late apoptotic cellular percentages increased also, with 7.72% for Col-NT and 8.42% for Col-DHT. As for the necrotic cells, reduced values were observed in comparison with the T_0_ contact point: 0.14% for Col-NT and 0.12% for Col-DHT (Table 5). This outcome could be due to the initial shock that cells tend to suffer after modifications in the culture media occur. 

#### 3.2.4. Hemocompatibility 

**Figure 15:**
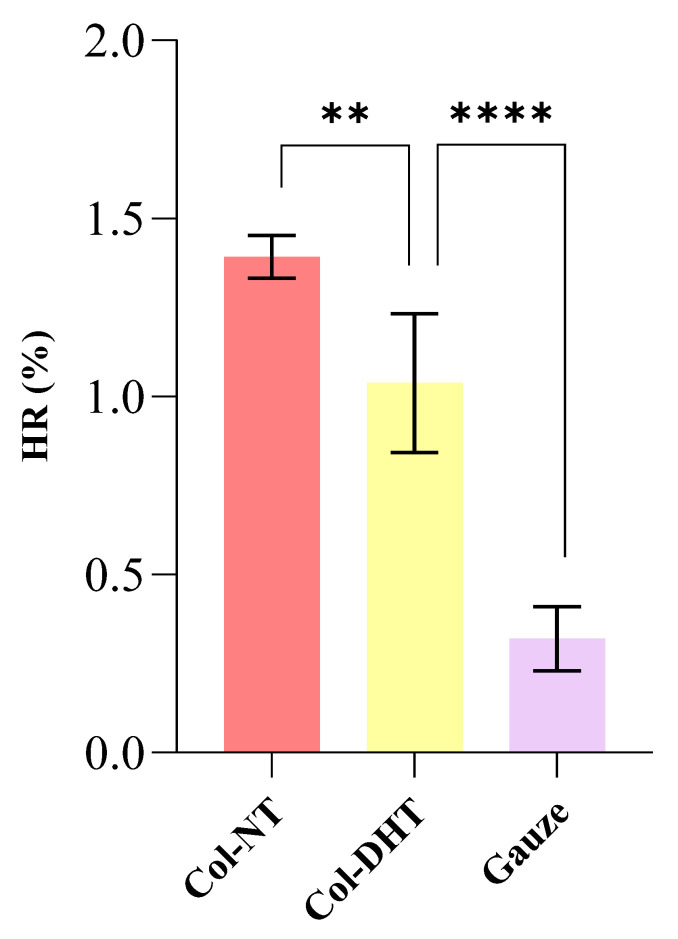
Evaluation of hemolysis performance of different materials in vitro. There was a significant difference in the hemolysis rate between Col-NT and Col-DHT sponges; ** = 0.0036; **** = < 0.0001.

The tested materials do not cause hemolysis. Generally, during hemolysis, red blood cells are lysed to release hemoglobin, which is then accumulated in the plasma. Hemolysis leads to various toxic biological effects on the body, so the compatibility of both implantable and open wound materials is necessary. The ideal biomedical product should be a non-hemolytic material with a hemolysis rate less than 5%, according to ASTM F 756–2000 requirements [45]. The results of this study showed that the hemolytic rate of Col-NT and Col-DHT was not higher than 5% (HR was 1.39 ± 0.06% and 1.04 ± 0.19%, respectively, *p* > 0.01) (Figure 15). These findings suggest that Col-DHT sponge may be a biomaterial with favorable compatibility with blood.

In correlation with the fluid uptake ability obtained for the Col-NT (35.61 ± 2.54) and Col-DHT (47.74 ± 1.04), the lower HR rates for the DHT sample can be explained by its ability to release hemoglobin being conditioned by the applied treatment, and, consequently, by the rigidity obtained for the fibrous scaffold. 

**Figure 16:**
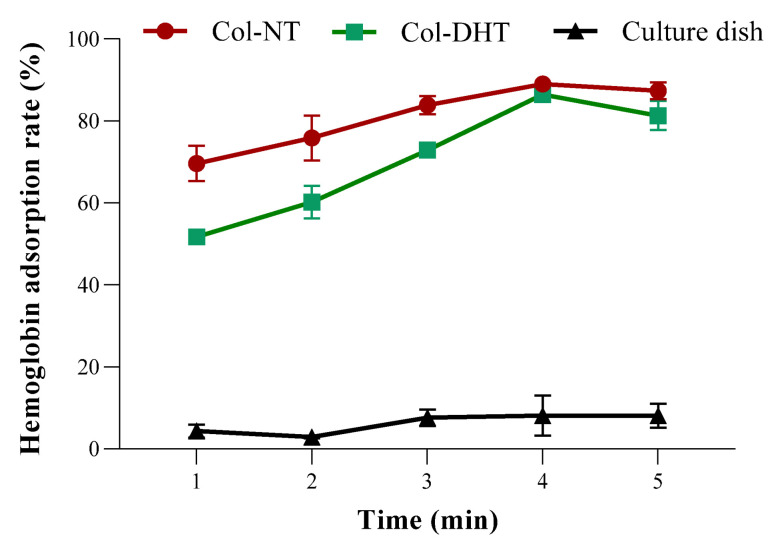
The hemoglobin adsorption of different collagen materials compared with culture dish.

The rate of hemoglobin adsorption by the collagen sponges is shown in Figure 16, where the concentration of hemoglobin in 10 μL of blood was set as 100%. Higher hemoglobin adsorption indicates that the blood was completely clotted. The hemoglobin adsorption rate for the culture dish was approximately 8.13%, which means that the blood clots were difficult to form on the glass within 5 min. A positive linear relationship between hemoglobin adsorption rates was observed in the first 4 min, with an increased adsorption rate for Col-DHT, though these values were lower values than for Col-NT. The findings indicate that Col-DHT started from a significantly lower absorption rate (*p* < 0.0001) and reached a coagulation effect similar to Col-NT after 4 min (*p* > 0.05).

#### 3.2.5. Biodegradability 

**Figure 17:**
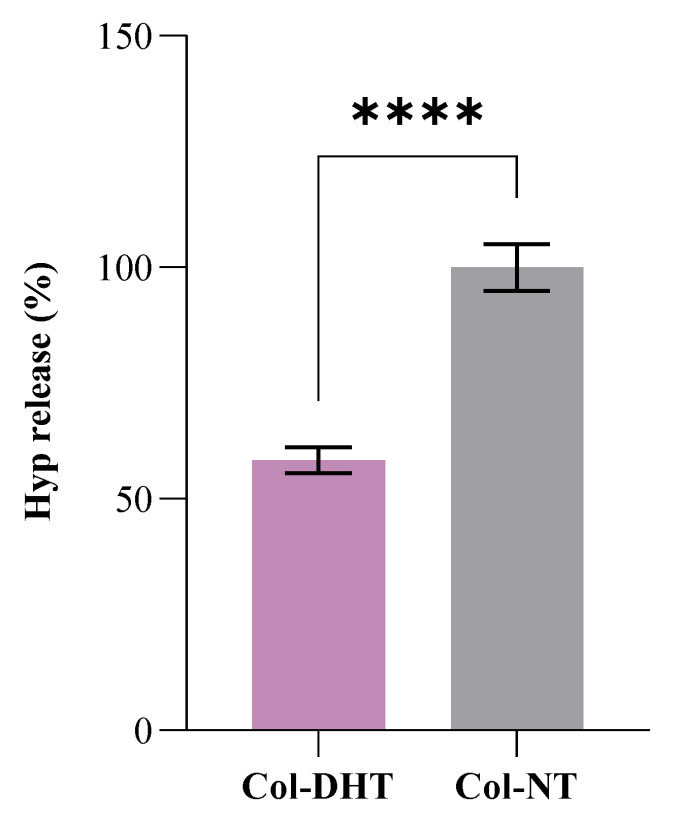
The biodegradation percentage expressed as hydroxyproline released from Col-NT and Col-DHT after 24 h of digestion; **** = < 0.0001.

Biomaterial biodegradation is vital for their use both as implantable materials and externally (e.g., in wound healing) because it affects cell growth, cell vitality and host response [46]. Biodegradability was expressed as a percentage of hydrolysis of collagen sponge exposure to bacterial collagenase and was analyzed by the hydroxyproline content released in the digestive juice. The degradation rate of the unmodified collagen sponge was set at 100%. Figure 17 showed the degradation percentage of the collagen sponges. The collagen sponge after crosslinking (Col-DHT) showed a degradation of 58.36 ± 2.86%, the reduction being significant (*p* < 0.0001). Therefore, Col-DHT was more resistant to the biodegradation of collagenase.

## 4. Discussion

Collagen is the most common natural polymer, being the most abundant protein on earth, and can be easily extracted from both animal and plant sources. Type I bovine collagen, extracted from tendons, skin and bones, is the most common form, being widely used as a biomaterial. The intermolecular crosslinking of collagen can be achieved by the esterification of carboxylic groups or by amide group formation, collagen being a protein with a high number of hydroxyl, carboxyl and amino groups.

Without crosslinking, collagen is frequently subject to problems related to its weak mechanical properties, cracking and high swelling ratio. Its stability can be increased with the formation of an extensive network of stable intermolecular crosslinks. Dehydrothermal (DHT) treatment, a physical crosslinking method, involves the exposure collagen to high temperatures for a certain period of time under a vacuum in order to remove the water and form new crosslinks. Removing the water from a collagen scaffold may cause damage to the original hydrogen bonds within the collagen. In this research, the most favorable DHT conditions were employed, with a highest temperature of 135 °C for 3 h.

In the present study, we successfully produced a crosslinked collagen sponge scaffold using DHT treatment with a highly porous structure. Porosity and density are two very important parameters that define porous scaffolds. The reduction in the porosity of the sponge upon DHT crosslinking was counteracted by an increase in density, which would favor cell growth, proliferation, and nutrient and metabolite exchange, due to the larger surface accessible to cells [47].

SEM analysis depicted the sample’s progression from a frail, fibrous and high porosity scaffold to a denser, rigid and condensed structure after the DHT treatment. As for the mechano-dynamic assessment, three domains can be identified: the first two following a conventional pattern, and the unusual third one, the latter probably associated with the progressive rupturing of the elongated fibers. The smallest feasible strain falling within the linear domain of the stress–strain curve was 65%. The thermally induced evolution of the main visco-elastic parameters of the scaffold material was observed in the domain between −50 … +100 °C, where the “melting” of the triple-helical structure of collagen occurs, at around 60 °C.

TG-DSC analysis of samples indicated similar behavior for both of them. Upon close inspection, the DSC curve obtained indicated the presence of a weak endothermic effect at 220 °C, corresponding to the transition from triple-helix to an accidentally twisted chain. This transition is specific to collagen, and occurs when hydrogen bonds are broken by rising temperatures, turning collagen into gelatin.

Evaluation of cellular biocompatibility in an in vitro stimulated tissue microenvironment provides great overview of the behavior of a medical device, especially in wound healing applications, where the physiopathology of the wound further complicates the affected site. The results obtained by MTT and LDH assays, performed after 24 h of sample contact with HEp-2 cells, indicate desirable biocompatibility for the sample Col-DHT, and decreased viability rates for Col-NT, further indicating the positive outcome of the DHT treatment. The monolayer exhibited better proliferation and adherence in the presence of the Col-DHT sample. The decreased viability for the sample Col-NT may be due to the different degradation process and the formation of small peptidic chains that suffocate cells and do not promote proper proliferation. As other authors have mentioned, biodegradation plays a critical role in obtaining a desirable 3D scaffold [48,49].

Cytokines are critical mediators that oversee and regulate immune and inflammatory responses via complex networks and serve as biomarkers for many diseases. Quantification of cytokines has significant value in both clinical medicine and biology, as the levels provide insights into physiological and pathological processes, especially in the development of novel medical devices of clinical interest. Cytokines and their clinical significance can be characterized from the perspective of their pro- and anti-inflammatory effects [50]. Proinflammatory cytokines including IL-1β, IL-6 and IFN-γ, facilitate inflammatory reactions and tend to stimulate immunocompetent cells. IL-6 has a duplicate role, acting as an anti-inflammatory cytokine, as well as inhibiting inflammation and suppressing immune cells. The cytokine interferon gamma (IFN-γ) is critical to both innate and adaptive immunity, and functions as the primary activator of macrophages, in addition to stimulating natural killer cells and neutrophils [34,37,40,51].

After exposing RAW murine macrophages to samples of Col-NT and Col-DTH, as well as *E. coli* and *S. aureus* (as positive controls), we observed lower levels of 1β and IL-6 for the collagen samples in comparison with the positive controls; as for IFN-γ, similar values for both collagenic scaffold samples were obtained, with decreased expression levels in comparison to *E. coli* and *S. aureus* controls. Conversely, stimulation with bacterial strains led to enhanced production of IL-1β, IL-6 and IFN-γ. A clear distinction between the two types of collagenic scaffolds was not observed, suggesting that the dehydrothermal reticulation did not impact cytokine expression in any way. Since IFN gamma is a pro-apoptotic effector [51], the results obtained correlate with the increased levels of early apoptosis cells observed during the flow cytometry assessment.

The evaluation of early/late apoptosis and necrosis provided great insights into the cellular behavior after treatment with the collagenic scaffolds. The evaluation at the point of initial contact presented higher rates of viability (over 90%). However, after 24 h, cellular viability decreased significantly, and early apoptosis occurred. These findings may indicate some limitations of the MTT and LDH assays; as we know, these provide colorimetric end-point measurements. Annexin V and Propidium Iodine labelling paint a more detailed picture of how cells are affected at the metabolic level for both early apoptosis pathways (extrinsic and intrinsic) [44], and false-positive results obtained by colorimetric methods may be due to incipient stages of early apoptosis, when metabolic functions are still optimal. 

The evaluation of hemocompatibility for scaffolds intended to be used as implantable devices is a crucial assay. The results obtained indicate a HR of 1.39 ± 0.06% for Col-NT and 1.04 ± 0.19% for Col-DHT. According to ASTM F 756–2000 requirements, both samples are non-hemolytic. These results correlate with the higher fluid uptake ability of the Col-DHT samples as compared to the Col-NT. Moreover, Col-DHT starts from a significantly lower absorption rate and reaches a coagulation effect similar to Col-NT after 4 min.

The biodegradability of samples was assessed using collagenase. Collagenase is an enzyme responsible for cleaving the triple helix. It specifically catalyzes the hydrolysis of peptide bonds with glycine [45]. The resistance to collagenase digestion of Col-DHT may be due to the retention of structural integrity due to its crosslinking and lower fluid absorption capacity [23]. Therefore, it was shown that the efficient crosslinking process significantly reduces the dissolution tendency. The collagen sponge after crosslinking (Col-DHT) showed a degradation of 58.36 ± 2.86% in comparison with Col-NT. Keeping in mind that, in vivo, untreated collagen is rapidly degraded, and its current use is therefore limited [52], this result is favorable and consistent with other authors’ findings [53] regarding the influence of crosslinking methods on the swelling ratio, mechano-dynamic characteristics and resistance to collagenase digestion. 

Compared with intact human skin, the mechanical and visco-elastic behavior of the studied scaffold registers modest values (with at least ten-fold less stress accommodated at the same applied strain) [54]. However, the role of the collagenous scaffold is not to directly replace the skin, but to progressively substitute it, by facilitating the healing processes in which is involved. 

## 5. Conclusions

An adapted DHT treatment with different conditions of time and temperature was employed to obtain the most appropriate collagenic scaffold that is intended to be used in due course as a medical device for wound healing. The formation of covalent crosslinking bonds was observed by analyzing the swelling ratio of collagen scaffolds in DI water and 6M aq. urea. The IR data confirm that the DHT treatment did not lead to the loss of the native triple helix structure of the collagen scaffold. SEM, TG-DSC and mechano-dynamic assessment further confirmed this and presented a detailed depiction of the scaffolds’ behavior. Furthermore, we focused on analyzing the biomimetic behavior of the collagenic scaffold attained. The satisfactory biocompatibility results obtained, with no inflammatory stimulation, decreased necrotic cells and low HR rates, suggest considerable scope for further studies. Higher percentages of cellular early apoptosis were observed. Collagenase degradation rates were consistent with literature reports regarding the influence of crosslinking methods on collagen scaffold degradation. 

Notwithstanding the favorable results obtained so far in this study, due to complex and comprehensive requirements from legislative parties, there is a long way to go before even attempting to market this type of product. Besides the biotechnological challenges, clinical trials and CE marking approval are among the ones that still lie ahead. 

## Figures and Tables

**Table 1:** Parameter variation (temperature, time and vacuum) during dehydrothermal reticulation of lyophilized collagen sponge.

	DAY 1			DAY 2		
Temperature (°C)	60	85	105	135	110	Room Temperature
Time (h)	1	6	over night	3	3	over night
Vacuum (mbar)	150	100	50	50	50	50

**Table 2:** Scaffold nitrogen and total protein content, humidity, swelling ratio and fluid uptake ability.

Sample	Nitrogen (%)	Total Protein (%)	Humidity (%)	Swelling Ratio		Porosity (%)	Density (%)	Fluid Uptake Ability
				AD	Urea			
Col-NT	14.77 ± 0.43	92.35 ± 2.71	13.23 ± 0.21	5.5 ± 0.12	15.5 ± 0.81	98.4 ± 0.05	17.4 ± 2.03	35.61 ± 2.54
Col-DHT	14.95 ± 0.10	93.44 ± 0.64	5.17 ± 0.07	2.9 ± 0.05	3.5 ± 0.30	93.3 ± 1.43	19.5 ± 1.19	47.74 ± 1.04

**Table 3:** Breakdown of the thermal behavior of the collagen scaffolds.

Sample	Mass Loss RT-160 °C	Mass Loss 160–700 °C	Endo	Exo I	ExoII	Exo III	Exo IV
Col-NT	10.19%	86.11%	87.3 °C	284.0 °C	331.2 °C	387–488 °C	631.1 °C
Col-DHT	9.09%	86.75%	87.3 °C	278.9 °C	325.3 °C	387–488 °C	624.7 °C

**Table 4:** Cellular behavior of HEK-293 T cells at the initial contact with tested scaffolds.

Sample	Viability (%)	Early Apooptotic Cells (%)	Late Apoptotic Cells (%)	Necrotic Cells (%)
Col-NT	90.19%	6.19%	2.80%	0.82%
Col-DHT	91.83%	4.95%	2.45%	0.76%

**Table 5:** Cellular behaviour of HEK-293 T cells after 24 h of contact with tested scaffolds.

Sample	Viability (%)	Early Apooptotic Cells (%)	Late Apoptotic Cells (%)	Necrotic Cells (%)
Col-NT	48.44%	43.70%	7.72%	0.14%
Col-DHT	50.35%	41.11%	8.42%	0.12%

## Data Availability

Not applicable.
